# Composition and Similarity of Bovine Rumen Microbiota across Individual Animals

**DOI:** 10.1371/journal.pone.0033306

**Published:** 2012-03-14

**Authors:** Elie Jami, Itzhak Mizrahi

**Affiliations:** 1 Department of Ruminant Science, Institute of Animal Sciences, Agricultural Research Organization, Bet Dagan, Israel; 2 Department of Molecular Microbiology and Biotechnology, The George S. Wise Faculty of Life Sciences, Tel Aviv University, Ramat-Aviv, Israel; Université Paris Sud, France

## Abstract

The bovine rumen houses a complex microbiota which is responsible for cattle's remarkable ability to convert indigestible plant mass into food products. Despite this ecosystem's enormous significance for humans, the composition and similarity of bacterial communities across different animals and the possible presence of some bacterial taxa in all animals' rumens have yet to be determined. We characterized the rumen bacterial populations of 16 individual lactating cows using tag amplicon pyrosequencing. Our data showed 51% similarity in bacterial taxa across samples when abundance and occurrence were analyzed using the Bray-Curtis metric. By adding taxon phylogeny to the analysis using a weighted UniFrac metric, the similarity increased to 82%. We also counted 32 genera that are shared by all samples, exhibiting high variability in abundance across samples. Taken together, our results suggest a core microbiome in the bovine rumen. Furthermore, although the bacterial taxa may vary considerably between cow rumens, they appear to be phylogenetically related. This suggests that the functional requirement imposed by the rumen ecological niche selects taxa that potentially share similar genetic features.

## Introduction

A significant proportion of domesticated animal species worldwide—the source of most meat and dairy products—are ruminants. Chief among these are dairy cattle. Ruminants are herbivores, and their digestive system allows them to absorb and digest large amounts of plant material. This capacity is of enormous significance to man, as ruminants essentially convert the energy stored in plant mass to digestible food products [1]. The ability to absorb and digest the plant material resides in the ruminants' foregut, the rumen, which is essentially a chambered anaerobic compartment. The rumen is inhabited by a high density of resident microbiota, consisting of bacteria, protozoa, archaea and fungi, which degrade the consumed plant materials [2]. The rumen microorganisms, of which bacteria are the most abundant and diverse (∼95% of the total microbiota [3]), ferment and degrade the plant fibers in a coordinated and complex manner which results in the conversion of plant materials into digestible compounds, such as volatile fatty acids and bacterial proteins. These, in turn, define the quality and composition of milk and meat and their production yields [4]–[6]. Hence, the rumen microbiota is essential to the animals' well being and productivity, and consequently mankind. Therefore an understanding of these complex microbial populations and their interactions is of great importance.

Several cultivation-free methods have been used to study rumen microbial communities in both domesticated and wild ruminants [4], [5], [6], [7], [8]. In a recent study, used denaturing gradient gel electrophoresis (DGGE) analysis to investigate the effect of rumen sampling location and timing on ruminal bacterial diversity [9]. That study revealed high similarity between samples taken from different locations and time points for each individual cow, but lower similarity between samples taken from different host animals [9]. Other studies have focused on changes occurring in the microbial community and gene expression following changes in diet [10], [11].

In a study examining the changes in ruminal bacterial communities during the feeding cycle, it was implied that cows fed the same diets can exhibit substantial differences in bacterial community composition [4]. Differences in rumen microbial composition were further emphasized in a recent metagenomic study exploring the ruminal fiber-adherent microbial populations of three steers, one of which had a microbiome and metagenome which were remarkably different from the other two [3]. These observations raise important and fundamental questions regarding ruminal bacterial populations, among them: How similar are the ruminal bacterial populations across individual animals fed the same diet in terms of composition, abundance and occurrence? Are there specific populations which are present across all individual rumens? If so, what is the extent and composition of these populations?

We addressed these questions by analyzing the compositions and similarities of bacterial populations from 16 animals' rumens using amplicon pyrosequencing of the V2 and V3 regions of the 16 S rRNA gene with a total of 162,000 reads, ∼10,000 reads per sample. We present a study characterizing the similarities in identity and abundance of the rumen bacterial populations across all samples, as well as of specific populations that were present in all rumen samples examined.

## Results

### Identity of the ruminal bacterial composition

**Figure 1:**
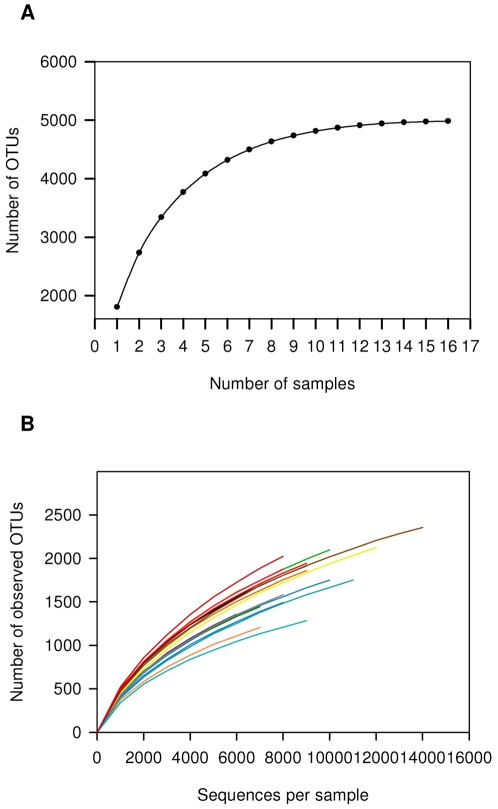
Rarefaction analysis for the assessment of OTU coverage. (**A**) Sample-based rarefaction curve showing the increase in OTU numbers as a function of the number of individuals sampled. Each added sample adds OTUs to the plot which has not yet been seen in previous samples. The curve becomes asymptotic as the OTU number saturates, and each sample adds an increasingly smaller number of new OTUs, indicating adequate coverage for the environment being tested. (**B**) Individual rarefaction curves for each rumen sample taken.

**Figure 2:**
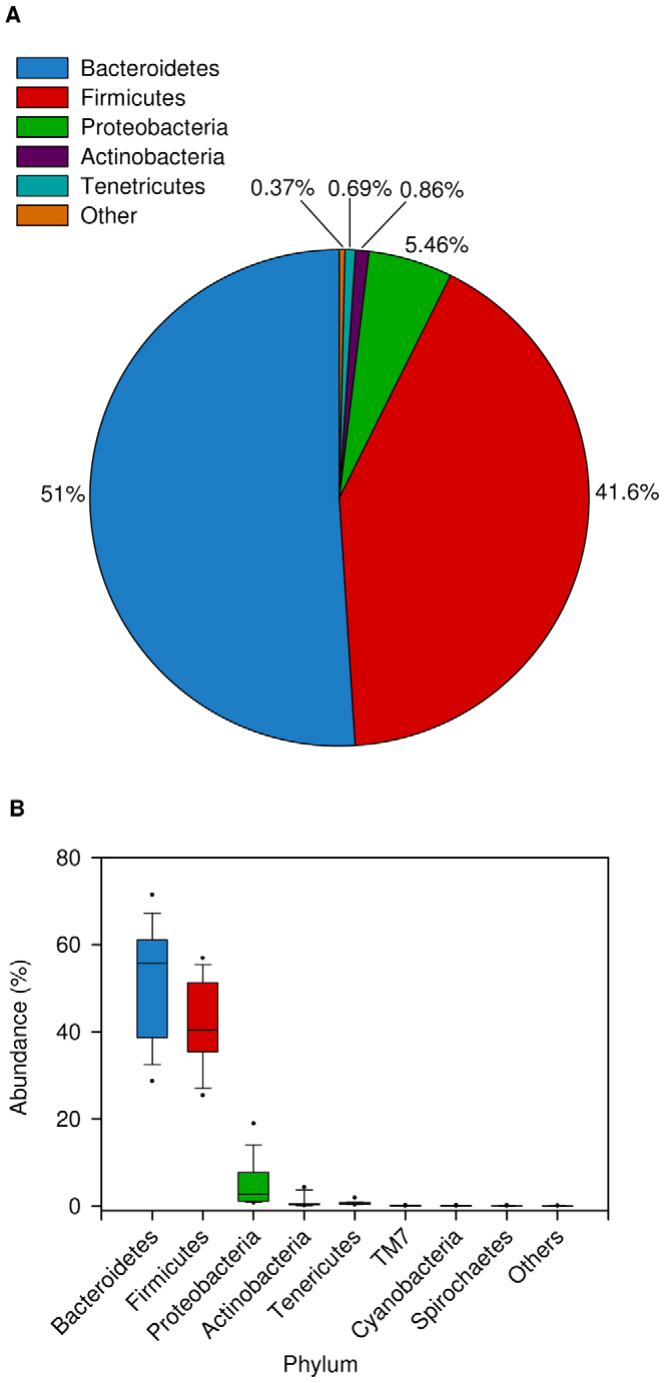
Composition and abundance of bacterial taxa, as determined by pyrosequencing of the 16 S rDNA gene. (**A**) Pie chart showing the average distribution of the phyla across all ruminal samples. (**B**) Box plot showing the relative abundance of each phylum, represented as percentage on the Y-axis. The boxes represent the interquartile range (IQR) between the first and third quartiles (25^th^ and 75^th^ percentiles, respectively) and the vertical line inside the box defines the median. Whiskers represent the lowest and highest values within 1.5 times the IQR from the first and third quartiles, respectively. Samples with a relative abundance of a given phylum exceeding those values are represented as points beside the boxes (color-coded).

We sampled the ruminal contents of 16 Holstein Friesian lactating cows fed the same diet ad libitum for several months and held under the same experimental conditions for 6 weeks. Samples were taken 1 h after feeding as described by Brulc *et al*. [3]. Microbial cells were separated from the rumen samples and their DNA was extracted using a protocol described by Stevenson and Weimer [6]. We then identified and characterized the overall ruminal bacterial composition as well as the taxa shared by all cows, by using bacterial tag-encoded amplicon pyrosequencing generated from the V2 and V3 regions of the 16 S rRNA gene. In total, 162,000 reads were generated with an average of 9587±2059 reads per sample. We used the QIIME pipeline [12] to filter the reads and for quality control, as well as for some of the data analyses. After filtering, quality control and chimera removal (see materials and methods), the total number of operational taxonomic units (OTUs) detected by the analysis reached 4986, with an average 1800±324 OTUs per rumen sample (an OTU was defined as a read sharing ≥97% nucleotide sequence identity) (Tables S1, S2). We performed a sample-based rarefaction test to assess whether our sampling and sequencing efforts provided efficient OTU coverage. After the tenth sample, the number of OTUs was saturated, as revealed by the asymptotic nature of the sample rarefaction curve (Figure 1A). Taxonomic assignment showed that the dominant ruminal bacterial phyla, summing to 93% of total bacterial reads, were Firmicutes and Bacteroidetes, representing 42% and 51% of total OTUs, respectively; 5.21% of the reads were attributed to the phylum Proteobacteria, 0.87% to Actinobacteria, and 0.68% to Tenericutes. Other phyla were also present but at lower percentages (Figure 2A). Examining each sample composition at the phylum level, we observed noticeable differences between individual cows reflected by changes in the abundance of Firmicutes, Bacteroidetes and Proteobacteria (Figure 2B).

### Core bacterial community shared by all cows

**Figure 3:**
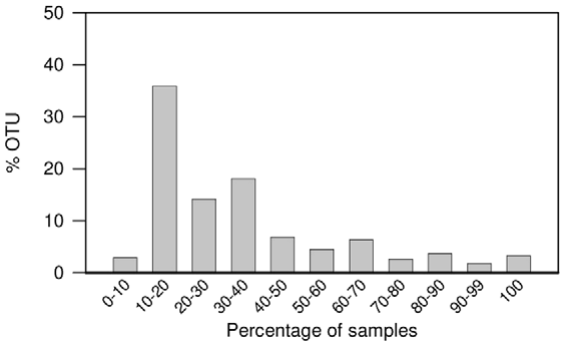
OTU occurrence across samples. Different OTUs were summed into categories according to their frequency of occurrence across different ruminal samples and binned accordingly, from OTUs shared by up to 10% of the samples to those shared by all samples. The X-axis represents the percentage of cows sharing a specific OTU. The Y-axis represents the percentage of OTUs found in each category.

**Figure 4:**
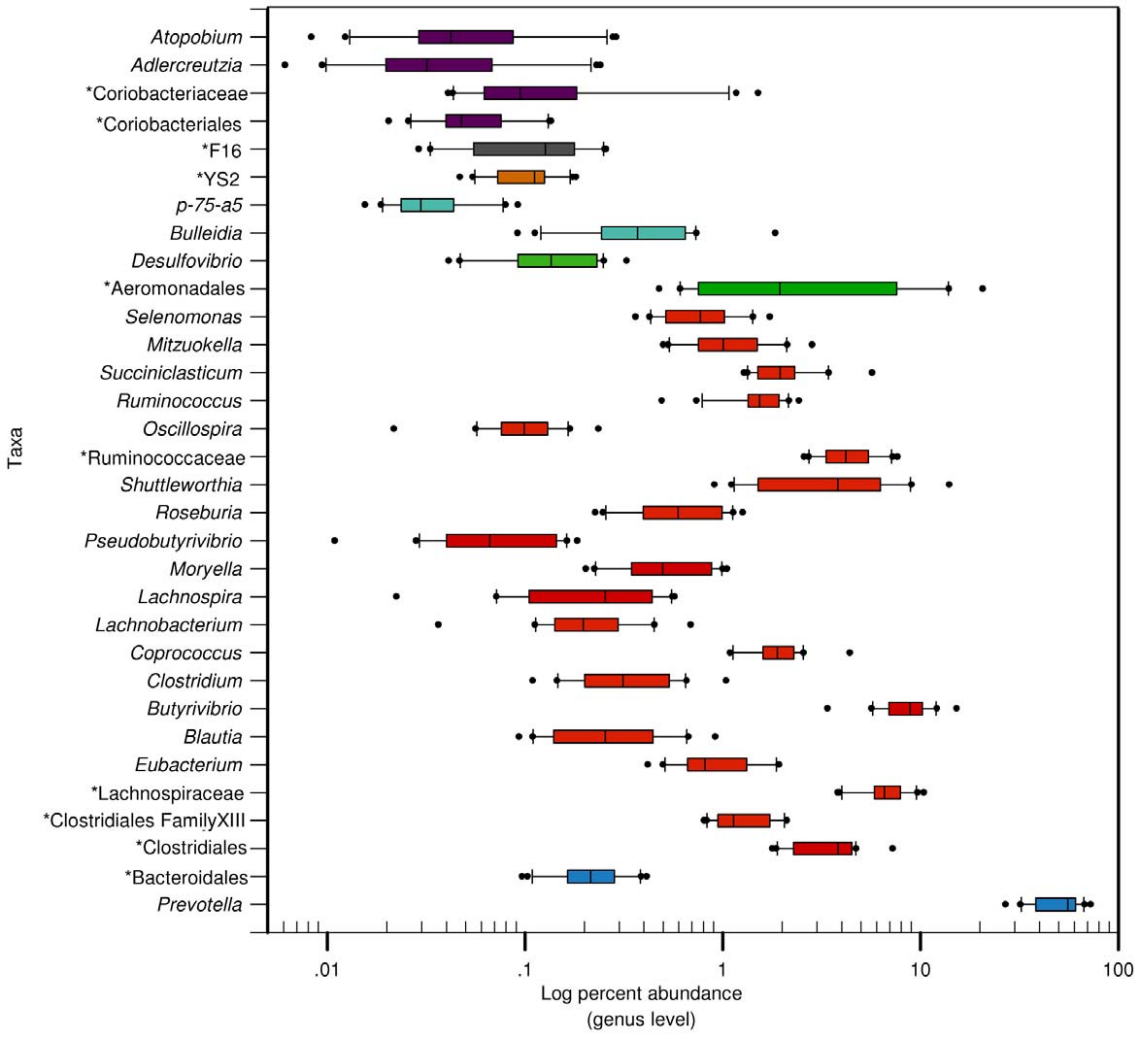
Shared genera and abundance across samples. Box plot showing the relative abundance of the bacterial genera shared by all samples, represented as log percentage on the X-axis. The boxes represent the interquartile range (IQR) between the first and third quartiles (25^th^ and 75^th^ percentiles, respectively) and the vertical line inside the box defines the median. Whiskers represent the lowest and highest values within 1.5 times the IQR from the first and third quartiles, respectively. Samples with a relative abundance of a given taxon exceeding those values are represented as points beside the boxes. The box color denotes the phylum of the genera: Bacteroidetes (blue), Firmicutes (red), Proteobacteria (green), Tenericutes (light blue), Cyanobacteria (orange), TM7 (gray), Actinobacteria (purple). Taxa not indentified at the genus level are identified by an asterisk and their highest taxonomic identification.

We analyzed our data for the distribution of each OTU across all samples. Occurrence of each OTU across the samples was evaluated and grouped into different categories according to prevalence. Figure 3 exhibits the percentage of OTUs shared by each sample occurrence category. This analysis revealed that 35% of the OTUs are present in only 10 to 20% of the samples and 14% are present in 20 to 30% of the samples, resulting in almost 50% of the OTUs being shared by a small proportion of the samples. This analysis also revealed a group with ∼4% of OTUs shared by all samples and a group of ∼2% shared by 90 to 99% of the samples. We next performed a genus-level analysis of the composition and abundance of the core bacterial community shared by 100% of the samples, and identified 32 genera that were shared by all samples (Figure 4). Some of the shared genera were highly abundant in the overall rumen bacterial community across the samples, such as the genus *Prevotella* which accounted for an average 52% of all rumen bacterial genera, while others, although shared by all of the samples, accounted for an average of 0.1% of the total rumen bacterial genera, such as the genus *Oscillospira* (Figure 4). Most of the shared genera varied in abundance across the samples. We further analyzed this core community at the species level (≥97%). This analysis revealed 157 OTUs shared by all samples with the highest representation from the following taxa: genus *Prevotella* (80 OTUs), family Lachnospiraceae (14 OTUs), genus *Butyrivibrio* (16 OTUs) (Table S3).

We also employed quantitative real-time PCR to monitor the presence and abundance of species belonging to genera which were not present in all samples but are considered to be important for the rumen ecosystem (Table S4). Most of the species examined were detected by the real-time analysis in all samples except *Ruminobacter amylophilus H18*, which was not found in all samples and exhibited very low abundance, just above detection level, when it was observed (Table S4).

### Similarity between cows

**Figure 5:**
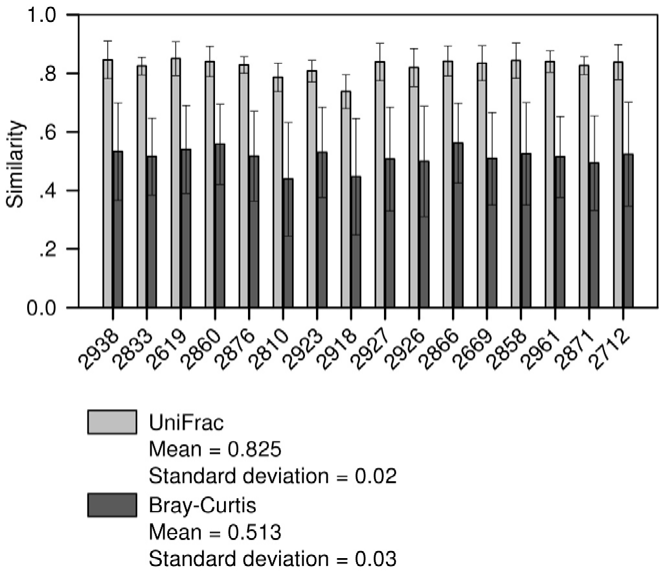
Pairwise similarity calculation. The average of pairwise comparisons of each sample to all others was calculated. The QIIME pipeline was used to compute the Bray-Curtis metric (gray bars) and the weighted UniFrac metric (black bars). The X-axis denotes the serial number of the cow from which the rumen sample was taken, and the Y-axis represents the degree of similarity: the closer the similarity to 1, the more similar the samples. At the bottom, average and standard deviation of each similarity metric calculated across all cow pairs are shown.

To assess the degree of similarity between the samples, we performed a pairwise similarity analysis in which the distances of each sample were paired and then averaged, giving the similarity of a specific sample to all others. To this end, we used the Bray-Curtis metric, as well as the weighted UniFrac metric which also measures the distance between communities based on their phylogenetic lineages (using the pyNAST QIIME implementation for sequence alignment and tree building [13]). Figure 5 shows the pairwise metric values for each individual cow and the average of each of the metrics for all possible cow pairs.

An average 51% similarity between each pair of samples was calculated by the Bray-Curtis metric, 82% using the weighted UniFrac metric. The datasets calculated by the two metrics were significantly different (t-test, *P*<0.001).

## Discussion

An understanding of the microbial ecosystem in the rumen is of great importance for the general study of microbial communities and their symbiosis with multicellular organisms, as well as for our everyday lives. A fundamental aspect in understanding any ecosystem is to identify its permanent and temporary residents. Therefore, the objectives of this study were to assess the general ruminal bacterial population in terms of total number of possible bacterial OTUs and taxa present, and to characterize the similarities across different cow rumens in terms of taxa's temporal and universal occurrence. To cover most of the possible bacterial OTUs found in the ruminal ecosystem of our study, we characterized the bacterial communities across a relatively large array of animal rumens and used tag-encoded amplicon pyrosequencing of the V2 and V3 regions of the 16 S rRNA gene at a depth of 10,000 reads per sample.

A sample-based rarefaction test on our data revealed that after the tenth sample (approximately 100,000 reads), most of the possible bacterial OTUs present in our overall rumen samples were covered. Note that this analysis is limited to the OTUs that were covered by the pyrosequencing procedure (PCR amplification, primers etc.). This finding confirms the conclusion of a recent in-silico study in which the collective microbial diversity in the rumen was examined by meta-analysis of all curated 16 S rRNA gene sequences deposited in the RDP database. The authors of that study estimated that 80,000 reads would cover all possible OTUs in the rumen [14]. Furthermore, in that study, the bacterial sequences were assigned to 5271 OTUs at the species level (≥97% similarity), which is also in agreement with our data in which a total of 4896 OTUs were assigned at ≥97% similarity.

An analysis of the prevalence of each OTU across all animals revealed that about 50% of them occur only in 0% to 30% of the animals sampled (Figure 3). Thus, despite the strict maintenance of similar experimental conditions, diet and sampling procedures, a large fraction of the OTUs occurred in only a small number of samples, contributing to the differences between the ruminal bacterial populations.

This was also seen in pairwise comparisons using the Bray-Curtis metric which indicated an average 51% similarity between the samples (Figure 5). However, when the weighted UniFrac metric was used to calculate the similarity between samples, an average value of approximately 82% was measured. The significant increase in similarity, compared to the Bray-Curtis metric, suggests that although a large number of OTUs differ between samples, they are phylogenetically related. This can be explained by the notion that the phylogenetically related OTUs have similar genetic profiles, enabling them to occupy proximal or similar ecological niches.

The average composition of the rumen bacterial community consisted mainly of the phyla Firmicutes and Bacteroidetes, 43% and 50% of all reads, respectively; the Proteobacteria accounted for 5.455% of the reads, and Actinobacteria and Tenericutes for 0.9% and 0.7%, respectively (Figure 2A). Interestingly, when we examined community composition for each individual sample, the ratios between the different phyla changed considerably among samples (Figure 2B): the Bacteroidetes were highly variable, with an abundance ranging from 26% to 70% of all reads. This observation concurs with a recent study of the human gut microbiome in which the Bacteroidetes also varied greatly among samples [15]. Similarly, the relative abundance of Proteobacteria varied considerably among samples, from 0.5% of all reads to as high as 20% in some samples. It is important to note that studies examining the phylum distribution in Holstein cows' 16 S rDNA clone libraries have observed distributions similar to some of the samples measured here [16].

An important finding of this work was that all sampled animals shared a group of bacterial taxa consisting of 32 genera which varied considerably in abundance across samples (Figure 4). The representation of these shared genera in the overall ruminal bacterial community was highly diverse, as low as 0.01% for some of the genera and up to 50% for others (Figure 4). It is tempting to speculate that even though some genera represent only a small fraction of the total rumen bacteria, the fact that they are shared by all animal rumens might indicate that they fill an important function in the rumen ecosystem or that they occupy a special ecological niche in the rumen; however, this speculation requires further examination, including a determination of their presence in animals at other growth stages and on different diets. Furthermore, the present study describes samples from the same breed and herd, and there is likely to be greater divergence between breeds, herds and geographical locations.

Interestingly, some bacterial taxa were absent from the core groups identified by pyrosequencing, even though some species of these taxa are considered crucial for fiber degradation in the rumen. Notably the phylum Fibrobacteres, which includes one of the main cellulolytic bacteria—*Fibrobacter succinogenes*—which is thought to be of great importance for rumen function, was found in only half of the samples. Several studies of the rumen microbiome have suggested that the abundance of this phylum, and in particular *F. succinogenes*, varies considerably across cows and diets. This was evident in a recent metagenomic study in which this phylum was completely absent from the fiber-adherent and total overall rumen microbiome [3]. In two recent studies, this phylum was shown to be influenced by fiber content in the diet: in one study examining 16 S clone libraries from the rumen, it was represented in 2 out of 647 clones in a low-fiber diet, increasing to 19 out of 620 clones in a high-fiber diet [17]; this trend was also observed in the other study using real-time PCR analysis [8].

To assess the possibility of dietary influence on the abundance of *Fibrobacter* in the current study, we pyrosequenced metagenomic DNA from a cow fed a higher content of fiber (50% instead of 30%). The higher-fiber diet led to 0.48% representation of the phylum Fibrobacteres, a 24-fold increase over the average 0.02% representation in the group of cows examined in this study (Figure S1). Nevertheless, the apparent absence of this phylum and other important rumen bacteria from some of the samples could be due to low abundance of these taxa, below the detection level of the pyrosequencing method. Therefore, to assess the possible presence of these rumen bacteria across the samples we used the more sensitive real-time PCR method. Indeed, when we measured the abundance of some rumen bacterial species known to be important for rumen function, including *F. succinogenes*, we found that most of these species exist in low numbers in all of the samples (Table S4). Bacterial species showing a relative abundance of less than 0.01% in our samples using real-time PCR were generally not detected by the pyrosequencing. Nonetheless, in most samples there was agreement in abundance between the pyrosequencing and real-time PCR results (Table S4). The genus *Succinovibrio* appeared to be consistently underrepresented when using the pyrosequencing method, with relative abundance values up to tenfold lower than the real-time PCR values, which could indicate either that the primers used for real-time PCR amplify other organisms or that the primers used for pyrosequencing are less adapted to amplification of this genus. This requires further investigation. The genus *Prevotella* was highly represented in our shared microbial community: it was the most abundant bacterial genus with an average 50% of all reads. This finding is consistent with a previous study by Stevenson and Weimer [9] in which several bacterial species were quantified in ruminal samples from two lactating cows using real-time PCR. That study reported the predominance of *Prevotella* members, which comprised 42% to 60% of the bacterial rRNA gene copies in the samples [6]. It is interesting to note that although relative quantification by real-time PCR was performed in that study, our pyrosequencing results were in the same range. The high abundance of this genus is interesting from an ecological point of view: it might be the result of a metabolic niche that is wide enough to be occupied by bacteria that have similar metabolic capabilities, due to genetic relatedness or to high genetic variability that enables members of this genus to occupy different ecological niches within the rumen. Indications from previous studies imply the latter, as members of this genus are considered to exhibit a remarkable degree of genetic diversity [18], [19], [20]. Nevertheless, these two possibilities need to be further examined and distinguished.

The changes in the overall rumen bacterial population, together with changes in the shared bacterial communities, are an important subject for further research. The nature of these variations and their effects on the animals warrant further careful characterization. For example, the stability of these differences over time in each individual animal should be further established, and their effects on rumen metabolic parameters and animal physiological characteristics should be examined as well.

The work presented here describes the composition of the overall bacterial communities of the rumen ecosystem, and their similarities and differences across individual cows fed the same diet. It also reveals a microbial community that was present in all rumen samples tested, and shows that while a large number of species are not shared by all samples, there is high phylogenetic similarity between the communities. These observations increase our understanding of this important microbial ecosystem, and raise new questions for further study.

## Materials and Methods

### Animal handling and sampling

The experimental procedures used in this study were approved by the Faculty Animal Policy and Welfare Committee of the Agricultural Research Organization (ARO) approval number IL-168/08, Volcani Research Center, and were in accordance with the guidelines of the Israel Council on Animal Care.

Israeli Holstein Friesian lactating cows (n = 16) were housed at the ARO's experimental dairy farm in Bet Dagan, Israel, in one shaded corral with free access to water. The cows were fed a diet consisting of 30% roughage and 70% concentrate as described in Table S5 ad libitum, provided once a day. The cows were kept on this diet for a few months prior to the experiment as this is the standard diet fed to lactating cows at the experimental farm. The corral holding the animals was specially designed to keep the animals as a group, thus maintaining normal herd behavior, while allowing individual feeding and monitoring: each cow had an electronic chip that opens its individual feeding area. The cows were kept in this facility on the above diet for 6 weeks prior to sample collection. The samples were taken 1 hour after the morning feeding: 500 ml of ruminal contents was collected via the cow's mouth using a stainless-steel stomach tube with a rumen vacuum sampler. The pH was determined immediately and was on average 6.51±0.37 across all samples. Samples were transferred to CO_2_-containing centrifuge bottles to maintain anaerobic conditions, and kept on ice. Immediately after collection, the ruminal samples were processed in the laboratory, located 100 m away.

After assessing several protocols for isolation of rumen microbes from the samples, we selected the one described by Stevenson and Weimer [6], as it exhibited the highest number of OTUs in an automated ribosomal intergenic spacer analysis (ARISA) (data not shown), as well as a good ability to detach the fiber-adherent bacteria, as reflected by enrichment of known fiber-adherent species quantified by real-time PCR analysis (data not shown). Isolation of the rumen microbial populations, including detachment of the fiber-adherent microbial populations and planktonic populations from the fibers, was performed with some minor modifications that included mixing the fiber-adherent microbial populations with the planktonic ones. Briefly, following 2 minutes of blender homogenization, the homogenate was centrifuged at 10,000 *g* and the pellet was dissolved in extraction buffer (100 mM Tris-HCl, 10 mM ethylenediaminetetraacetic acid [EDTA], 0.15 M NaCl pH 8.0): 1 g of pellet was dissolved in 4 ml of buffer and incubated at 4°C for 1 hour, as chilling has been shown to maximize the release of particle-associated bacteria from ruminal contents [6]. The suspension was then centrifuged gently at 500 *g* for 15 minutes at 4°C to remove ruptured plant particles while keeping the bacterial cells in suspension [21]. The supernatant was then passed through four layers of cheesecloth and centrifuged (10,000 *g*, 25 minutes, 4°C), and the pellets were kept at −20°C until DNA extraction.

### DNA extraction

The DNA extraction was performed as described by Stevenson and Weimer [6]. Briefly, cell lysis was achieved by bead disruption with phenol followed by phenol/chloroform DNA extraction. The final supernatant was precipitated with 0.6 vol isopropanol and resuspended overnight in 50 to 100 µl Tris-EDTA buffer, then stored at 4°C for short-term use, or archived at −80°C.

### Real-time PCR

Quantitative real-time PCR analysis was performed to investigate the relative abundance of specific bacterial species through amplification of their copy of the 16 S rRNA gene using the primers shown in Table S6 [6], [22]. A standard curve was generated for each bacterial strain selected. By amplifying a serial twofold dilution of gel-extracted PCR products obtained by the amplification of each amplicon, we generated individual standard curves suitable for the quantification of each bacterial strain individually. A standard curve was also generated for the total bacterial 16 S rRNA gene in the samples by amplifying 10-fold dilutions of the gel-purified PCR product of one rumen sample. The standard curves were obtained using four dilution points, and were calculated using Rotorgene 6000 series software (Qiagen, Germany). Subsequent quantifications were calculated with the same program using the standard curve generated in each run (equating to one bacterial species), and at least one known purified product dilution used for the standard curves was added to each quantification reaction in order to assess the reproducibility of the reactions. All obtained standard curves met the required standards of efficiency (R^2^>0.99, 90%>E>115%). Real-time PCR was performed in a 10-µl reaction mixture containing 5 µl Absolute Blue SYBR Green Master Mix (Thermo Scientific), 0.5 µl of each primer (10 µM working concentration), 3 µl nuclease-free water and 2 µl of 10 ng DNA templates. Amplification involved one cycle held at 95°C for 15 minutes for initial denaturation and activation of the hot-start polymerase system, and then 40 cycles at 95°C for 10 seconds followed by annealing for 15 seconds at 60°C and extension at 72°C for 20 seconds. To determine the specificity of amplification, a melting curve of PCR products was monitored by slow heating from 60 to 99°C (alternating 1°C increments with holding for 10 seconds), with fluorescence collection at 1°C intervals. Quantification of the selected bacteria was performed by dividing the specific bacterial count obtained for each bacterium, using the appropriate set of primers, by the total bacterial count obtained by amplification with the universal bacterial primers.

### 454 tag amplicon pyrosequencing and data analyses

454 amplicon pyrosequencing of the ruminal DNA samples was performed by the Research and Testing Laboratory (Lubbock, TX) using primers covering the 103- to 530-bp region of the 16 S rRNA gene sequence which corresponds to the V2 and V3 regions (107 F: 5′-GGCGVACGGGTGAGTAA-3′ and 530 R: 5′-CCGCNGCNGCTGGCAC-3′). The tagging and sequencing protocol was as described by Dowd *et al*. [23].

Data quality control and analyses were mostly performed using the QIIME pipeline [12]: 256,000 raw reads were assigned to their designated rumen sample using the split_library.py script which also performs quality filtering based on length (<200 bp) and quality of the reads, resulting in 162,000 reads of 351 bp on average (all read parameters per sample are listed in Table S2). The next step was to align the obtained sequences to define OTUs, in order to eventually assign taxonomy to them. Different OTU-generation methods have been reported to give different estimates of OTU number [24]. Therefore, we used three different clustering methods for OTU generation: UCLUST [25], ESPRIT-tree [26] and CD_HIT_OTU [27] (Table S1), which have been proven to generate satisfactory and comparable numbers of OTUs [24]. These analyses showed that the CD_HIT_OTU provides a significantly higher number of OTUs compared to the UCLUST method. The ESPRIT-tree method resulted in a slightly lower number of OTUs which was not significantly different from the UCLUST results (*P*>0.05). Therefore, the UCLUST method was selected to further analyze the data as it was the better well-adapted to the QIIME pipeline. The degree of similarity between sequences was defined as ≥97% to obtain OTU identity at the species level. Next we used the Chimera Slayer algorithm [28] for chimeric sequence removal. OTUs which clustered only one or two reads were manually removed. These processes resulted in 153,000 reads for analysis.

After constructing an OTU table, taxonomy was assigned using the BLAST algorithm and the reference database found at: http://blog.qiime.org designated “most recent Greengenes OTUs”. In parallel, phylogeny was calculated using the pyNAST [29] algorithm to create a tree which would enable the generation of UniFrac similarity measurements between samples. For the similarity measurement between the bacterial communities in the samples, two similarity indices were used: a Bray-Curtis comparison between samples according to both the presence and absence of OTUs and the abundance of OTUs between the samples, and the UniFrac metric, which uses the phylogenetic tree of the created OTUs to compare the phylogenetic closeness of the bacterial community between samples.

## Supporting Information

Figure S1 **Pie chart showing the phylum distribution of one rumen sample taken from a cow fed a 50% fiber diet.**
(EPS)

Table S1 Comparison of different clustering methods and number of OTUs generated by each method.
(PDF)

Table S2 Number of reads and mean length of reads per sample that were used for the analysis (after quality filtering and removal of chimeras, singletons and doubletons).
(PDF)

Table S3 Taxonomic identification of the core OTUs (97% similarity) found in 100% of the samples and the number of OTUs associated with each specific taxon.
(PDF)

Table S4 Comparative analysis of real-time PCR and pyrosequencing. Comparative analysis of real-time PCR results from five specific bacterial species and the abundance observed for their respective genera using pyrosequencing. The mean and standard error of the mean (S.E.M) were also calculated for both methods. Pearson correlation was calculated between the pyrosequencing and real-time PCR results for each taxon.
(PDF)

Table S5 Formulated ingredients (g/kg DM) of the basic total mixed rations.
(PDF)

Table S6 PCR primers used for detection of rumen bacteria in this study by real-time PCR [6], [22].
(PDF)
