# Supplementary material for: Composition and Similarity of Bovine Rumen Microbiota across Individual Animals
Source: PLoS One. 2012 Mar 14;7(3):e33306. doi: 10.1371/journal.pone.0033306 (PMC3303817; doi:10.1371/journal.pone.0033306)
Supplement: Table S1 — Comparison of different clustering methods and number of OTUs generated by each method. (PDF) [file pone.0033306.s002.pdf]

Table S1. Comparison of different clustering methods and number of OTUs generated by each method.

	UCLUST	CD_HIT	ESPRIT-tree
Total raw OTU number	16094	18356	20344
Total number of chimeric OTUs identified	2929	3047	2420
Total OTU number after singleton and doubleton removal	4986	6222	4861
