# Supplementary material for: Composition and Similarity of Bovine Rumen Microbiota across Individual Animals
Source: PLoS One. 2012 Mar 14;7(3):e33306. doi: 10.1371/journal.pone.0033306 (PMC3303817; doi:10.1371/journal.pone.0033306)
Supplement: Table S2 — Number of reads and mean length of reads per sample that were used for the analysis (after quality filtering and removal of chimeras, singletons and doubletons). (PDF) [file pone.0033306.s003.pdf]

Table S2. length and number of the reads per cow sampled after quality filtering, chimeric sequences removal and singletons-doubletons removal.

Cow #	Mean read length	Number of reads per animal
2918	332 +/- 72	9940
2866	336 +/- 76	12052
2669	348 +/- 83	9690
2858	342 +/- 81	9125
2961	334 +/- 85	11532
2871	342 +/- 72	7868
2712	339 +/- 82	9536
2938	335 +/- 81	10165
2833	342 +/- 74	6858
2619	339+/- 81	8367
2860	336 +/- 83	10241
2876	338 +/- 77	8135
2810	341 +/- 73	7322
2923	331 +/- 71	8803
2927	336 +/- 81	8537
2926	334 +/- 86	15225
**Average**	**338 +/- 80**	**9587 +/- 2059**
