# Supplementary material for: Composition and Similarity of Bovine Rumen Microbiota across Individual Animals
Source: PLoS One. 2012 Mar 14;7(3):e33306. doi: 10.1371/journal.pone.0033306 (PMC3303817; doi:10.1371/journal.pone.0033306)
Supplement: Table S3 — Taxonomic identification of the core OTUs (97% similarity) found in 100% of the samples and the number of OTUs associated with each specific taxon. (PDF) [file pone.0033306.s004.pdf]

Table S3. identification of the core OTUs found in 100% of the samples

Taxonomic identification	Number of core OTUs (97% similarity) associated with a specific taxonomic identification
(Order) *Bacteroidales* sp.	1
(Genus) *Prevotella* sp.	80
(Order) *Clostridiales* sp.	8
(Family)*Clostridiales FamilyXIII.IncertaeSedis* sp.	7
(Family) *Lachnospiraceae* sp.	14
(Genus) *Butyrivibrio* sp.	16
(Genus) *Coprococcus* sp.	1
(Genus) *Eubacterium* sp.	2
(Genus) *Lachnobacterium* sp.	1
(Genus) *Lachnospira* sp.	1
(Genus) *Moryella* sp.	1
(Genus) *Shuttleworthia* sp.	4
(Family) *Ruminococcaceae* sp.	10
(Genus) *Ruminococcus* sp.	4
(Family) *Veillonellaceae* sp.	4
(Order) *Aeromonadales* sp.	1
(Family) *F16* sp.	1
(Family) *Coriobacteriaceae* sp.	1
Total	157
