# Supplementary material for: Composition and Similarity of Bovine Rumen Microbiota across Individual Animals
Source: PLoS One. 2012 Mar 14;7(3):e33306. doi: 10.1371/journal.pone.0033306 (PMC3303817; doi:10.1371/journal.pone.0033306)
Supplement: Table S4 — Comparative analysis of real-time PCR and pyrosequencing. Comparative analysis of real-time PCR results from five specific bacterial species and the abundance observed for their respective genera using pyrosequencing. The mean and standard error of the mean (S.E.M) were also calculated for both methods. Pearson correlation was calculated between the pyrosequencing and real-time PCR results for each taxon. (PDF) [file pone.0033306.s005.pdf]

Table S4. Real time PCR quantification results of 4 species known for their important metabolic functions in the rumen compared with the abundance observed for their respective genera using pyrosequencing.

Sample #	*Fibrobacter succinogenes S85*	*Fibrobacter* genus (pyrosequencing)	*Megasphera elsdenii T81*	*Megasphera* genus (pyrosequencing)	*Ruminobacter amylophilus H18*	*Ruminobacter* genus (pyrosequencing)	*Succinivibrio dextrinosolvens 22b*	*Succinovibrio* genus (pyrosequencing)
1	0.010%	0.030%	0.058%	0.028%	0.000%	0.000%	0.280%	0.219%
2	0.008%	0.000%	0.009%	0.008%	0.000%	0.000%	0.352%	0.164%
3	0.003%	0.010%	0.007%	0.000%	0.000%	0.010%	0.795%	0.673%
4	0.029%	0.081%	0.004%	0.000%	0.000%	0.000%	0.250%	0.094%
7	0.001%	0.000%	0.046%	0.041%	0.000%	0.000%	0.281%	0.180%
8	0.007%	0.000%	0.095%	0.121%	0.000%	0.000%	0.288%	0.090%
9	0.004%	0.010%	0.003%	0.000%	0.001%	0.010%	0.238%	0.022%
10	0.001%	0.009%	0.007%	0.000%	0.000%	0.009%	0.252%	0.087%
11	0.002%	0.000%	0.050%	0.041%	0.000%	0.000%	0.290%	0.134%
14	0.081%	0.101%	0.017%	0.000%	0.000%	0.000%	0.275%	0.191%
15	0.010%	0.000%	0.007%	0.019%	0.000%	0.000%	0.383%	0.047%
16	0.005%	0.023%	0.012%	0.000%	0.000%	0.000%	0.209%	0.115%
17	0.003%	0.000%	0.067%	0.116%	0.000%	0.000%	0.445%	0.407%
19	0.005%	0.000%	0.040%	0.011%	0.000%	0.000%	0.300%	0.232%
20	0.002%	0.020%	0.003%	0.000%	0.001%	0.031%	0.148%	0.000%
21	0.004%	0.000%	0.008%	0.006%	0.001%	0.049%	0.121%	0.060%
Mean	0.011%	0.018%	0.027%	0.024%	0.000%	0.007%	0.306%	0.169%
S.E.M	0.005%	0.008%	0.007%	0.010%	0.000%	0.004%	0.038%	0.042%
Pearson correlation	0.881		0.888		0.879		0.877	
