# Supplementary material for: Composition and Similarity of Bovine Rumen Microbiota across Individual Animals
Source: PLoS One. 2012 Mar 14;7(3):e33306. doi: 10.1371/journal.pone.0033306 (PMC3303817; doi:10.1371/journal.pone.0033306)
Supplement: Table S5 — Formulated ingredients (g/kg DM) of the basic total mixed rations. (PDF) [file pone.0033306.s006.pdf]

Table S5. Formulated ingredients (g/kg DM) of the basic total mixed rations.

Ration composition of 30% fiber diet of the 16 lactating cows		Ration composition of 50% fiber diet of one dairy cow	
Wheat silage	100	Bran	93
Oat hay	80	Soy molassa	100
Wheat straw	-	Straw	100
Sunflower straw	-	Hay	269
Corn silage	100	Sunflower shell	77
Clover hay	23	lactose	24
Soybean hulls	78	F gluten	54
Soybean meal (solvent extracted)	22	growth mix	39
Ground corn grain	129	Pima cotton	77
Ground barley grain	87	corn sillage	77
Ground wheat grain	44	water	34
Whole cottonseeds	20	Sunflower hulls	56
Corn gluten Feed	96		
Corn distillers dry grain	89		
Rapeseed meal	38		
Whey solids	36		
$NaHCO_3$	7.4		
NaCl	6		
$CaCO_3$	9		
[2]Ca-LCFA	14		
Soy molassa	17		
Urea	4		
Trace mineral + vitamin mixture[1]	0.6		

[1]The trace minerals + vitamins mix contained (g/ kg DM): Zn, 24; Fe, 24; Cu, 12.8; Mn, 24; I, 1.44; Co, 0.32; Se, 0.32; Vit. A 16,000,000 IU; Vit. D_3, 3,200,000 IU; Vitamin E, 48,000 IU.

[2] Calcium salts of long-chain fatty acids
