# Supplementary material for: Composition and Similarity of Bovine Rumen Microbiota across Individual Animals
Source: PLoS One. 2012 Mar 14;7(3):e33306. doi: 10.1371/journal.pone.0033306 (PMC3303817; doi:10.1371/journal.pone.0033306)
Supplement: Table S6 — PCR primers used for detection of rumen bacteria in this study by real-time PCR [6], [22]. (PDF) [file pone.0033306.s007.pdf]

Table S6. PCR primers used for detection of rumen bacteria in this study by Real-Time PCR

Bacteria	Primers	Reference
Fibrobacter succinogenes S85	GCGGGTAGCAAACAGGATTAGA	[9]
	CCCCCGGACACCCAGTAT	
Megasphaera elsdenii T81	AGATGGGGACAACAGCTGGA	[9]
	CGAAAGCTCCGAAGAGCCT	
Ruminobacter amylophilus H18	CTGGGGAGCTGCCTGAATG	[9]
	GCATCTGAATGCGACTGGTTG	
Succinivibrio dextrinosolvens 22b	CGTCAGCTCGTGTCGTGAGA	[9]
	CCCGCTGGCAACAAAGG	
HAD-domain bacteria	ACTCCTACGGGAGGCAGCAGT	[22]
	GTATTACCGCGGCTGCTGGCAC	
